# ﻿A comparative cytogenetic study of *Hypsibarbusmalcolmi* and *H.wetmorei* (Cyprinidae, Poropuntiini)

**DOI:** 10.3897/compcytogen.17.107703

**Published:** 2023-09-15

**Authors:** Sudarat Khensuwan, Weerayuth Supiwong, Chatmongkon Suwannapoom, Phichaya Buasriyot, Sitthisak Jantarat, Weera Thongnetr, Nawarat Muanglen, Puntivar Kaewmad, Pasakorn Saenjundaeng, Kriengkrai Seetapan, Thomas Liehr, Alongklod Tanomtong

**Affiliations:** 1 Department of Biology, Faculty of Science, Khon Kaen University, Muang, Khon Kaen 40002, Thailand; 2 Faculty of Interdisciplinary Studies, Khon Kaen University, Nong Khai Campus, Muang, Nong Khai 43000, Thailand; 3 Department of Fishery, School of Agriculture and Natural Resources, University of Phayao, Muang, Phayao 56000, Thailand; 4 Faculty of Science and Technology, Rajamangala University of Technology Suvarnabhumi, Mueang Nonthaburi, Nonthaburi 11000, Thailand; 5 Department of Science, Faculty of Science and Technology, Prince of Songkla University, Pattani 94000, Thailand; 6 Division of Biology, Department of Science, Faculty of Science and Technology, Rajamangala University of Technology Krungthep, Bangkok 10120, Thailand; 7 Department of Fisheries, Faculty of Agricultural Technology, Sakon Nakhon Rajabhat University, Sakon Nakhon 47000, Thailand; 8 Faculty of Science and Technology, Mahasarakham Rajabhat University, Muang, Maha Sarakham 44000, Thailand; 9 School of Agriculture and Natural Resources, University of Phayao, Tumbol Maeka, Muang District, Phayao Province, 56000 Thailand; 10 Institute of Human Genetics, University Hospital Jena, Jena 07747, Germany

**Keywords:** Fish cytogenetics, Cyprinidae, microsatellites, chromosomes

## Abstract

Cyprininae are a highly diversified but demonstrably monophyletic lineage of cypriniform fishes. Here, the karyotype and chromosomal characteristics of *Hypsibarbusmalcolmi* (Smith, 1945) and *H.wetmorei* (Smith, 1931) were examined using conventional, nucleolus organizing regions (NORs) and molecular cytogenetic protocols. The diploid chromosome number (2n) of *H.malcolmi* was 50, the fundamental number (FN) was equal to 62, and the karyotype displayed 8m + 4sm + 38a with NORs located at the centromeric and telomeric positions of the short arms of chromosome pairs 1 and 2, respectively. 2n of *H.wetmorei* was 50, FN 78, karyotype 14m + 14sm + 22a with the NORs at the telomeric position of the short arm of chromosome pair 2. 2n and FN in males and females were identical. Fluorescence *in situ* hybridization using different microsatellite motifs as probes also showed substantial genomic divergence between both studied species. In *H.wetmorei*, (CAG)_n_ and (CAC)_n_ microsatellites accumulated in the telomeric regions of all chromosomes, while in *H.malcolmi*, they had scattered signals on all chromosomes. Besides, the (GAA)n microsatellites were distributed along all chromosomes of *H.malcolmi*, but there was a strong hybridization pattern in the centromeric region of a single pair in *H.wetmorei*. These cytogenomic difference across the genomes of these *Hypsibarbus* Rainboth, 1996 species are markers for specific evolutionary differentiation within these two species.

## ﻿Introduction

**Figure 1.**
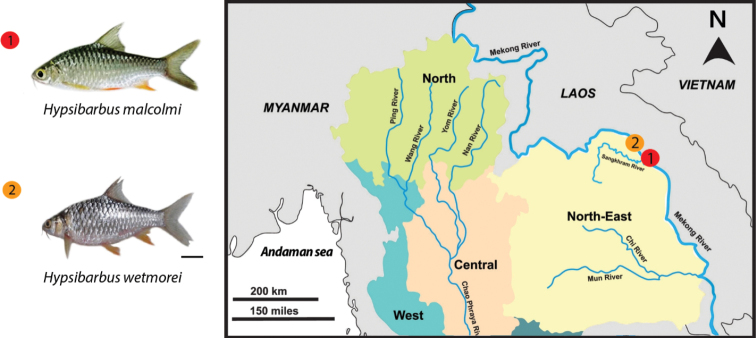
Collection sites of *Hypsibarbusmalcolmi* (1) and *H.wetmorei* (2) in the Mekong River, North-East Thailand (18°17'48.2"N, 104°00'16.9"E and 18°17'59.9"N, 104°00'09.5"E). Scale bar for fish: 1 cm.

The Cyprininae are the largest subfamily of the family Cyprinidae, which are the most diverse group of freshwater fish worldwide. This subfamily currently includes 33 genera, with 228 species being widely distributed in the freshwater systems of Eurasia (Fricke et al. 2023). *H.malcolmi* (Smith, 1945) and *H.wetmorei* (Smith, 1931), two yet understudied examples of Cyprininae, are widely distributed in Thailand’s rivers Mekong, Songkhram, Chao Phraya and Sirindhorn peat swamp forest. The two species mentioned have been shown to be the most similar to each other in external morphology and coloration (Fig. 1) and may be considered a species complex (Rainboth 1996). In addition, these two species are placed in the tribe of Poropuntiini on the phylogenetic reconstruction proposed by Yang et al. (2015). The classification of these fishes has been extremely difficult (Nelson 1994; Rainboth 1996; Ruber et al. 2007; Fang et al. 2009; Yang et al. 2010).

The diploid chromosome number of *H.malcolmi* and *H.wetmorei* has been reported as 2n = 50, but the karyotype and NF of *H.malcolmi* seem to be different (Magtoon and Arai 1989; Piyapong 1999; Donsakul and Magtoon 2002; Donsakul et al. 2007; Chantapan 2015; Khensuwan et al. 2023). Cytogenetics has become an important tool for fish classification, including cyprinids (Yang et al. 2015). Hereby, an important characteristic is the localization of nucleolus organizer region(s) (NOR(s)) as an inter- and intraspecies-specific marker for cytotaxonomic studies; NORs have been used for studying phylogenetic relationships between Cyprinids (Amemiya and Gold 1988; Galetti Jr 1998; Almeida-Toledo et al. 2000).

Classical and molecular cytogenetics play a crucial role in elucidating evolutionary patterns in cyprinid fish, especially in cases when species exhibit conserved diploid numbers. The abundance and chromosomal location of specific repetitive DNAs (microsatellites) change significantly between genomes of closely related species, and these variations are generally species-specific (Pereira et al. 2013). For example, the dinucleotides (CA)15 and (GA)15 accumulated exclusively in telomeric and subtelomeric chromosomal regions, corroborating findings from other fish groups studied to date (Terencio et al. 2013; Xu et al. 2013; Yano et al. 2014; Oliveira et al. 2015, 2018; Pucci et al. 2016). Otherwise, the genome of the wolf fish *Hopliasmalabaricus* (Bloch, 1794), with 12 different microsatellite repeats ((A)30, (C)30, (CA)15, (GA)15, (GC)15, (CAC)10, (CAA)10, (CAG)10, (CAT)10, (GAG)10, (TAA)10 and (CGG)10) showed strong hybridization signals at subtelomeric and heterochromatic regions of several autosomes, with a varied amount of signal on the sex chromosomes (Cioffi et al. 2011). So, in our study using trinucleotides (CAG)10, (GAA)10 and (CAC)10 observed patterns in the dynamics of the *Hypsibarbus* Rainboth, 1996 genome. Such microsatellites are predominantly located in the heterochromatic regions (telomeres, centromeres and sex chromosomes) of fish chromosomes, where a significant fraction of repetitive DNA is localized (Yüksel and Gaffaroğlu 2008; Knytl et al. 2013; Saenjundaeng et al. 2018, 2020; Phimphan et al. 2020; Saenjundaeng et al. 2020; Wang et al. 2022; Kentachalee et al. 2023). Single short repeats (SSRs) are short motifs that are repeated across the genome and consist of one to six nucleotides (Cioffi and Bertollo 2012; López-Flores and Garrido Ramos 2012). By supporting the correct pairing of the DNA double strand and preventing replication errors such as the creation of loops or other structures, they contribute to the stability of DNA molecules (Schueler et al. 2001). Furthermore, repeated DNAs play an essential role in speciation, sex differentiation, and biodiversity (Vicari et al. 2005; Cioffi et al. 2009; Sember et al. 2018).

The present study includes in-depth cytogenetic analyses of *H.malcolmi* and *H.wetmorei* (not a hybrid), comprising conventional Giemsa- and Ag-NOR staining and fluorescence in situ hybridization (FISH) approaches with chromosomal mapping of several repetitive DNA classes (microsatellites).

## ﻿Material and methods

### ﻿Animals

Individuals of *H.malcolmi* (12♂ and 6♀) and *H.wetmorei* (8♂ and 8♀) were collected in the Mekong River basin (Thailand) (Fig. 1). Fish were transferred to the laboratory and identified according to the morphological criteria of Rainboth et al. (2012). Experiments were performed in accordance with ethical protocols, with anesthesia using clove oil (Eugenol 3%) prior to the euthanasia, as approved by the Ethics Committee of Khon Kaen University (Record No. IACUC-KKU-105/63). The specimens were deposited in the fish collection of the Cytogenetic Laboratory, Department of Biology, Faculty of Science, Khon Kaen University (Thailand). DOI: dx.doi.org/10.17504/protocols.io.36wgq3r8klk5/v1.

### ﻿Chromosome preparation and NOR staining

Mitotic chromosomes were obtained from the anterior kidney following the drop onto microscopic slides and the air-dry method to visualize the chromosomes (Bertollo 2015). Conventional staining was performed using 5% Giemsa for 8 min (Khensuwan et al. 2023). In addition, the distribution of NORs was visualized according to the standard protocol using silver (Ag) staining (Howell and Black 1980). The slides were then sealed with cover slips and incubated at 60 °C for 5 minutes. After that, they were soaked in distilled water until the cover slips were separated. The glass slides were stained with 5% Giemsa for 1 minute.

### ﻿Probe preparation and FISH experiments

FISH experiments were performed under high stringency conditions (Yano et al. 2017) to classify microsatellite sequences, specifically (CAG)10, (GAA)10, and (CAC)10. These sequences were directly labeled by Cy3 at the 5'end during synthesis (Sigma, St. Louis, MO, USA) as described by Kubat et al. (2008). FISH was performed under stringent conditions and hybridization occurred overnight in a moist chamber at 37 °C (Sassi et al. 2023). Chromosomes were counterstained with 4',6-Diamidino-2-phenylindole dihydrochloride (DAPI, 1.2 μg/ml) mounted in antifade solution (Vector, Burlingame, CA, USA,).

### ﻿Image processing

At least 20 metaphase spreads per individual were analyzed to confirm the diploid number, karyotype structure, NORs and FISH data. Images were captured using an Axioplan II microscope (Carl Zeiss Jena GmbH, Germany) with CoolSNAP and processed using Image Pro Plus 4.1 software (Media Cybernetics, Silver Spring, MD, USA). Chromosomes were classified according to centromere position as metacentric (m), submetacentric (sm) and acrocentric (a) (Levan et al. 1964). For the chromosomal arm number (FN; fundamental number) m+sm were scored as bi-armed while a as mono-armed.

## ﻿Results

### ﻿Chromosome number, karyotype and fundamental number

**Figure 2.**
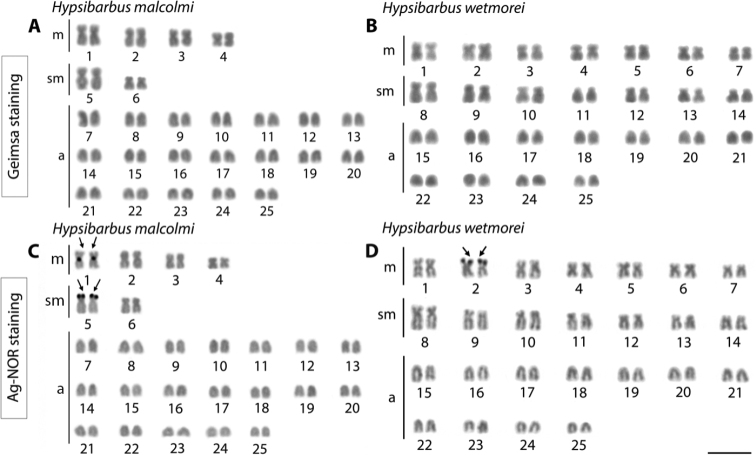
Karyotypes after conventional Giemsa (**A, B**) and NOR staining (arrows) (**C, D**) of *Hypsibarbusmalcolmi*, 2n = 50 (**A, C**) and *H.wetmorei*, 2n = 50 (**B, D**). Scale bar: 5 μm.

Cytogenetic analysis of *H.malcolmi* revealed 2n = 50 and FN = 62 in both sexes with a karyotype composed of 8 metacentric, 4 submetacentric and 38 acrocentric chromosomes (Fig. 2A). On the other hand, although *H.wetmorei* also showed 2n = 50, its FN was equal to 78, given its karyotype being composed of 14 metacentric, 14 submetacentric and 22 acrocentric chromosomes.

**Table 1.** Available cytogenetic data for *Hypsibarbus* species.

Species	2n	FN	Karyotype	Locality	NORs site	References
*Hypsibarbuslagleri* Rainboth, 1996	50	74	4m + 20sm + 26a	Thailand	-	Donsakul et al. 2002
*Hypsibarbusmalcolmi* (Smith, 1945)	50	64	10m + 4sm + 36a	Thailand	-	Donsakul et al. 2007
	50	62	8m + 4sm + 38a	Thailand	1, 5	Khensuwan et al. 2023
	50	62	8m + 4sm + 38a	Thailand	1, 5	Present study
*Hypsibarbusvernayi* (Norman, 1925)	50	58	6m + 2sm + 4st + 38a	Thailand	-	Donsakul et al. 2002
*Hypsibarbuswetmorei* (Smith, 1931)	50	70	12m + 8sm + 6st + 24a	Thailand	-	Magtoon and Arai 1989
	50	74	12m + 12sm + 4st + 22a	Thailand	2	Piyapong 1999
	50	74	12m + 12sm + 2st + 24a	Thailand	-	Donsakul et al. 2002
	50	82	10m + 14sm + 8st + 18a	Thailand	6	Chantapan 2015
	50	78	14m + 14sm + 22a	Thailand	2	Khensuwan et al. 2023
	50	78	14m + 14sm + 22a	Thailand	2	Present study

### ﻿NOR- staining and FISH results

While *H.malcolmi* had two pairs of NOR-bearing chromosomes, *H.wetmorei* had only one such pair. In the first, Ag-NOR regions were located at the centromeric and telomeric positions of the short arms on metacentric pairs 1 and 5 (Fig. 2C), while in *H.wetmorei* they were restricted to the telomeres on the short arms of pair 2 (Fig. 2D).

**Figure 3.**
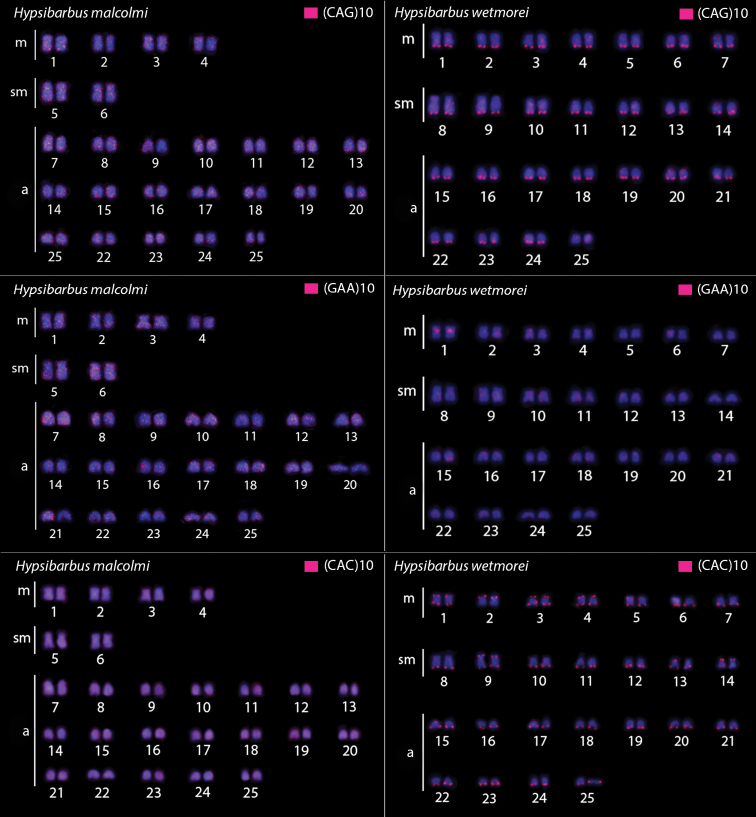
Hybridization patterns with microsatellite probes (CAG)_10_, (GAA)_10_ and (CAC)_10_ (red signals) on metaphase plates of *Hypsibarbusmalcolmi* and *H.wetmorei*. Chromosomes were counterstained with DAPI (blue). Scale bar: 5 μm.

In *H.wetmorei* the (CAG)n and (CAC)n microsatellites accumulated in the telomeric regions of all chromosomes, while *H.malcolmi* had scattered signals along all 50 chromosomes. (GAA)n presented strong signals in the centromeric regions of a single chromosomal pair in *H.malcolmi*, but a scattered distribution among all chromosomes in *H.wetmorei* (Fig. 3).

## ﻿Discussion

Our study has characterized populations of *H.malcolmi* and *H.wetmorei* by classical and molecular cytogenetics. For both species, diploid number and other features described in the scientific literature were confirmed (Magtoon and Arai 1989; Piyapong 1999; Donsakul and Magtoon 2002; Donsakul et al. 2007; Chantapan 2015; Khensuwan et al. 2023). 2n = 50 was reported for both species, as in the whole Cyprinidae lineage, which has been cytogenetically investigated so far and all of them exhibit a remarkable 2n conservation of 50 chromosomes. But with distinct karyotype organization in different species and populations. However, such a preserved 2n is clearly linked to substantial intrachromosomal changes, as also demonstrated by the discrepant NOR- and microsatellite patterns obtained in this study, emphasizing the importance of structural rearrangements in the evolution of this family, such as chromatin duplications/deletions, pericentric inversions, transpositions, and translocations (Pereira et al. 2011; Saenjundaeng et al. 2020; Khensuwan et al. 2023). Such rearrangements can also be observed as distinct patterns of NOR and microsatellite distribution among populations.

The position of NOR was consistent with the previous report for both species, with two pairs in *H.malcolmi* and one in *H.wetmorei*. The occurrence of multiple NORs in fish was considered to be apomorphic, whereas a single pair of NORs is considered to be plesiomorphic (Gold and Amemiya 1986). In species with multiple NORs, interindividual variation is common suggesting that transposition of rDNA genes from one chromosome to another may occur (reviewed in Phillips and Rab 2001). For example, *Salvelinusnamaycush* (Walbaum, 1792) (Phillips and Ihssen 1989), *S.alpinus* (Linnaeus, 1758) (Phillips et al. 1988) and *Salmotrutta* Linnaeus, 1758 (Castro et al. 1994) found differences in the number of NORs. Previous investigations in Cyprinidae have shown that almost all NOR sites correspond to active 18S rDNA loci (Khensuwan et al. 2023). The 18S rDNA is clustered with the 5.8S and the 25S rDNAs in plants, although only the first composes the small subunit of ribosomes (Goffová and Fajkus 2021). In fish genomes, 18S rDNA is usually located at the terminal position on chromosomes (Sochorová et al. 2018). This was also observed in both studied *Hypsibarbus* species, in addition to a centromeric site at the first chromosome pair in *H.malcolmi*. Although it is known that the terminal position of this rDNA facilitates the arrangement of the NOR in the interphase nucleus, centromeric NORs were found in the karyotypes of several species (e.g. Barth et al. 2013; Sassi et al. 2021), including species that only harbor a single rDNA locus (e.g. Sing and Barman 2013). Indeed, the number of NORs presented in the genome varies by species, and the rDNA content of NORs can differ between individuals of the same species and even between cells within an individual (Stults et al. 2008, 2009). Because ribosomal gene arrays are extremely repetitive, they are prone to homologous recombination (HR), creating unstable areas that could favor chromosomal rearrangements (Kobayashi 2008). The pattern observed in *H.malcolmi* could be a hint at a paracentric inversion of the short arm of chromosome 1. Normally, the NORs/18S rDNA is commonly found in a terminal location inside chromosomes (Sochorová et al. 2018), except for *H.malcolmi* located in the centromeric region. It is also remarkable that a large variety of karyotype re-organization occurs among populations.

The instability of repetitive regions of the genome can also be observed by microsatellites. These small repetitive motifs have been shown to stall and reverse replication forks, and to be hotspots of chromosomal double strand breaks in model organisms (Pelletier et al. 2003; Kerrest et al. 2009; reviewed in Gadgil et al. 2017). In fish, they are also accumulated in sex chromosomes (Schemberger et al. 2019). Closely related species can have very distinct patterns of microsatellite accumulation, as observed in the two species of *Hypsibarbus* here studied. Such discrepancy is more notable when comparing the (CAG)n, (GAA)n and (CAC)n motifs that are dispersed in the *H.malcolmi* genome but accumulate in the telomeres of *H.wetmorei*. Microsatellite motifs had a preferential accumulation in heterochromatic regions (reviewed in Cioffi and Bertollo,2012). However, the majority of the (CAG)n, (GAA)n and (CAC)n microsatellite sequences in *H.malcolmi* showed a scattered pattern on chromosomes, without a specific relationship with heterochromatic regions. Nevertheless, the (CAG)n, (GAA)n and (CAC)n motif presented a strong accumulation pattern in the telomeric regions of *H.wetmorei*. Also, (CA)n, (GC)n and (TA)n microsatellites accumulated in telomeric regions in both *H.malcolmi* and *H.wetmorei* (Khensuwan et al. 2023). It is known that triplet sequences are able to stabilize by harping on some alternative structures generated from errors of DNA polymerase (Sinden 1999); their presence at telomeres can be related to some repair mechanism. Repeated elements have been shown to be good tools for studying biodiversity, since they can “escape” from selection pressure that works on non-repetitive regions, making them evolutionary markers for detecting recent evolutionary changes (Cioffi et al. 2012; Garrido Ramos 2017; Moraes et al. 2017). Although the “Poropuntiinae” are thought to have diverged from other cyprinids about 37 Myr ago (Yang et al. 2021), recent changes in the genomes of those related species can have occurred, given the discrepant patterns of microsatellites and NOR herein observed, as in previous investigations as well (Khensuwan et al. 2023).

## ﻿Conclusions

This study applied conventional and molecular cytogenetics to study the karyotypes and chromosomal characteristics of *H.malcolmi* and *H.wetmorei*. Both species present similar morphology and a conservative 2n = 50. However, they can be distinguished based on their chromosomal morphology, NORs sites and repetitive DNAs, such as (CAG)n, (GAA)n and (CAC)n, showed specificities in their distribution among species, thus being shown as good markers and promoters of specific genomic differentiation inside the genus.
